# Physiological and Selective Attention Demands during an International Rally Motor Sport Event

**DOI:** 10.1155/2015/638659

**Published:** 2015-03-19

**Authors:** Anthony P. Turner, Hugh Richards

**Affiliations:** Institute for Sport, PE & Health Sciences, University of Edinburgh, Edinburgh EH8 8AQ, UK

## Abstract

*Purpose*. To monitor physiological and attention responses of drivers and codrivers during a World Rally Championship (WRC) event. *Methods*. Observational data were collected from ten male drivers/codrivers on heart rate (HR), core body (*T*_core_) and skin temperature (*T*_sk_), hydration status (urine osmolality), fluid intake (self-report), and visual and auditory selective attention (performance tests). Measures were taken pre-, mid-, and postcompetition day and also during the precompetition reconnaissance. *Results*. In ambient temperatures of 20.1°C (in-car peak 33.9°C) mean (SD) peak HR and *T*_core_ were significantly elevated (*P* < 0.05) during rally compared to reconnaissance (166 (17) versus 111 (16) beats·min^−1^ and 38.5 (0.4) versus 37.6 (0.2)°C, resp.). Values during competitive stages were substantially higher in drivers. High urine osmolality was indicated in some drivers within competition. Attention was maintained during the event but was significantly lower prerally, though with considerable individual variation. *Conclusions*. Environmental and physical demands during rally competition produced significant physiological responses. Challenges to thermoregulation, hydration status, and cognitive function need to be addressed to minimise potentially negative effects on performance and safety.

## 1. Introduction

Competitive motor sport requires high levels of physical and cognitive performance whilst experiencing significant environmental, physical, and psychological demands. These include physical work against considerable gravitational and physical forces in confined spaces; climatic conditions; compulsory safety clothing (fire-protective under-clothing, overalls, balaclavas, helmets, boots, and gloves); proximity to hot engines; pressure to succeed; significance of winning; fear of failure; competitors; observation by spectators and media; and increased information load and distraction. Determining the impact of the demands is important to assist maintaining or enhancing physiological and psychological components of performance and to maximize safety and competition outcome. Relevance of such study extends beyond motor sport to other domains (military, police, and emergency services) where skilled performers with good fitness complete cognitively challenging performances in clothing and conditions that contribute to demands.

Heart rates ranging 130–200 beats·min^−1^ have been recorded across a range of different motor sport disciplines, conditions and simulated versus real competition scenarios [1–5]. Previously, higher heart rates in competition were interpreted as primarily “psychoemotional” in nature [5]; however measurement [3] and estimation [6] of energy expenditure as 5–13 times higher than resting (METS) illustrate that drivers must work hard to perform accurate physical and cognitive tasks under physiological conditions similar to some other sports. Furthermore, in some motor sports competition duration can be considerably longer, spanning multiple days, which increases the probability that central and peripheral fatigue will impact performance.

The effects of heat and dehydration, which in real life setting are usually combined, have been the subject of much research in military [7], occupational [8], and sports settings [9–12]. Current understanding about dehydration, hyperthermia and performance has evolved from “critical threshold” theories, since research has shown performers successfully enduring higher temperatures and greater dehydration during real competitive events [9–12]. For example, ironman triathletes and sailing crews have experienced average core temperatures of 38.1°C (23°C ambient) and 39.2°C (32°C ambient), respectively, during performances with some individual values in excess of 40°C without incident [13, 14]. However, evidence from endurance performances with less cognitive processing demands and little constraint on clothing cannot be directly compared to motor sport. Although equivocal, recent evidence from controlled laboratory studies suggests even moderate levels of dehydration (1-2% body mass) can have a significant negative impact on cognitive function [15, 16] with less effect from hyperthermia alone. Roberts and Cole [17] found that 5 minutes exercise wearing police body armour produced changes in cognitive function (decreased executive and increased nonexecutive). However, the student participants were completing unfamiliar activities and tasks in which they had no specialized ability or training. Consequently findings do not accurately reflect how expert performers may be able to maintain complex performances where training and skill may offset performance decrements by focusing resources on critical aspects and utilizing specific strategies.

Currently, laboratory research that has sought to isolate distinct effects of heat and dehydration does not have the ecological validity necessary to transfer knowledge to real world applications which requires a more applied approach to research. Morley and colleagues [8] found that 50–120 minutes of exercise, wearing firefighters' protective equipment in 33–35°C heat, produced a rapid rise in core body temperature and a delayed drop in cognitive performance an hour or more following exercise. Interestingly, whilst cognitive tasks completed through audio recordings partially resemble firefighters' cognitive demands of radio communications, the participants were a mix of firefighters and other volunteers. The experience and motivations of volunteer samples are radically different to individuals involved in military operations, emergency response or professional competitive sport, and this may significantly impact on performance capacity.

Thus, the current study aimed to characterize some of the changes in physiological (heart rate, thermoregulation and hydration) and selective attention markers observed in a group of drivers and codrivers during a high-pressure, international motor sport event—a round of the World Rally Championship (WRC). It was hypothesised that there would be significant elevations in heart rate, skin and core temperature, during rally compared to reconnaissance. Furthermore, it was hypothesised that there would be significant differences in urine osmolality and selective attention within and between rally and reconnaissance.

## 2. Method

### 2.1. Participants

All ten male subelite performers (5 driver/codriver pairs; Mean (SD) age, 30 (9) years; Height, 1.76 (0.05) m; Mass, 75.9 (11.2) kg) selected onto an international driver development programme (Pirelli Star Driver) signed informed consent to participate. The study was approved by the Research Ethics Subcommittee of the School of Education, University of Edinburgh. Medical questionnaires were screened for contraindications associated with ingested core temperature sensors [18]. Permission was provided by the medical delegate for WRC from the world governing body (Federation Internationale de l'Automobile) and the event Chief Medical Officer.

### 2.2. Rally Event

Rally is a classification of motor sport involving driver and codriver (navigator) driving a series of stages in the fastest cumulative time, on a variety of surfaces. Cars, resembling showroom models, are turbo-charged, stripped down for weight and strengthened to withstand high speeds (0–100 km·h^−1^ in <3 s; peak > 200 km·h^−1^) and impacts of corners and jumps. This study took place during one round of The World Rally Championship (WRC), the premier competition comprising normally 13 events per year, in locations around the world in sometimes extreme climates. The event in Portugal spanned six days, including two days of reconnaissance making pace notes driving the route at restricted speeds in a normal road car without any safety clothing or helmets, followed by an interval day with mechanical testing and media commitments. Competitive driving then lasted two and a half days with 18 timed “special stages” (ranging 1 to >40 km each), separated by hundreds of kilometres driving on open, public roads at legal speeds. Cars completed approximately 3 or more stages consecutively before returning for short (30 minute) servicing. In WRC winning margins have been as little as 0.1 seconds after three days and several hundred stage kilometres, so enhancing any relevant performance factor could potentially influence outcome. Note that access to participants was completely restricted to one reconnaissance and one competition day within this single event and potential experimental disruption to performance had to be minimised. This was one event of only 6 that this development program entailed, and these drivers had very minimal precompetition testing days in the specific and very expensive rally cars, further adding to the pressure of the event.

Continuous measurement of heart rate (HR), core (*T*_core_) and skin temperature (*T*_sk_) was completed during one reconnaissance and one competition day. Participants were also assessed at early morning (pre), during (mid) and at the end of the day (post) for urine osmolality (*U*_osm_), selective attention and self-reported fluid intake (volume and content).

### 2.3. Heart Rate, Core and Skin Temperatures

A lightweight (total ~ 80 g) ambulatory monitoring system (Equivital EQ01, Hidalgo Ltd., UK) was used to record (1 Hz) *T*_core_ (from ingestible pill sensor—VitalSense, Mini Mitter, Philips Respironics, The Netherlands), and *T*_sk_ (°C) and HR (beats·min^−1^) (from skin thermistor and sensors in elasticated chest strap). Pill sensors were ingested 3 hours before recording to ensure good reliability and validity of measurement was obtained [18].

Peak in-car temperature was measured using lightweight battery operated thermometers (Model 810–210, Electronic Temperature Instruments Ltd., UK; range −50 to 70°C, accuracy ±1°C, resolution ±0.1°C). Competition restrictions precluded more sophisticated in-car measurement.

### 2.4. Urine Osmolality

With limited access to participants during the days of monitoring and no immediate access prior to the event, hydration markers such as changes in body mass and blood markers were excluded. Therefore hydration status was estimated from urine osmolality using the refractive index, a recognised field-based measurement [19, 20]. Midstream samples (~20 mL) from pre- (first morning), mid-, and postreconnaissance and rally days were analysed using a portable refractometer (Osmocheck, Vitech Scientific Limited, West Sussex, UK).

### 2.5. Selective Attention

Two subtests of the Test of Everyday Attention [21], used previously to assess attentional processing changes in military survival environment [22], were selected as they correspond specifically to some of the performance demands of rally (listening to audio communications and map reading) and did not require specific language proficiency or educational attainment:* Map search*—assessed visual selective attention requiring a 120 second search for 80 target symbols distributed on a coloured map.* Elevator counting with distraction*—assessed auditory-verbal working memory requiring participants to mentally count target tones whilst ignoring distracting tones. To minimise practice and learning effects participants were familiarised with tests the day before the first measurement. Three equivalent versions of both subtests were used and repeated tests with the same version were more than 76 h apart.

### 2.6. Statistical Analysis

Data was recorded for 10 participants during the reconnaissance and 8 participants on the rally. Data from one participant, taking nonprescription medication for cold-symptoms, was omitted from urine analysis, and data from one participant unable to discriminate tonal differences was omitted from auditory attention scores. Descriptive statistics (Mean (SD) and peak values) were calculated across 6 h on reconnaissance day and 9 h on rally day. Values were also calculated for HR during the rally special stages. Comparison between drivers and codrivers was restricted to qualitative interpretation of the descriptive statistics because of small subgroup size.

Mean and peak values for HR, *T*_core_ and *T*_sk_ during the rally and reconnaissance were not normally distributed and were compared using Wilcoxon matched pairs test. Effect sizes for Wilcoxon matched pairs test were calculated as *r* by dividing *Z* score by number of observations. A one-way ANOVA with repeated measures was conducted over the time-points for *U*_osm_ and attention scores, with Bonferroni adjusted post hoc comparisons. Statistical significance was set a priori at alpha <0.05. Effect sizes were calculated using partial eta squared (partial *η* ^2^) square-rooted to give correlation coefficients (*r*) [23]. Comparison for effect sizes were made in line with Hopkins [23]; 0.1–0.3 as small, 0.3–0.5 as moderate, 0.5–0.7 as large and 0.7–0.9 as very large.

## 3. Results

### 3.1. Conditions

Mean ambient temperature during the reconnaissance was 19.4°C (peak 23°C, mean relative humidity 70%) and during the rally was 20.1°C (peak 24.2°C, mean relative humidity 67%). Cabin temperatures peaked at 36.9°C and 33.9°C in reconnaissance and rally, respectively. Reconnaissance driving involved 124.1 km of special stages at normal speeds (capped at 90 km·h^−1^) and 125.7 km of road-driving. Rally driving involved 135.1 km (6 stages) at competitive speeds and 300.1 km of road-driving. The stages were very similar in distance (range 20.2–22.7 km), each lasting approximately 15 minutes in duration.

### 3.2. Heart Rate, Core and Skin Temperatures

**Figure 1:**
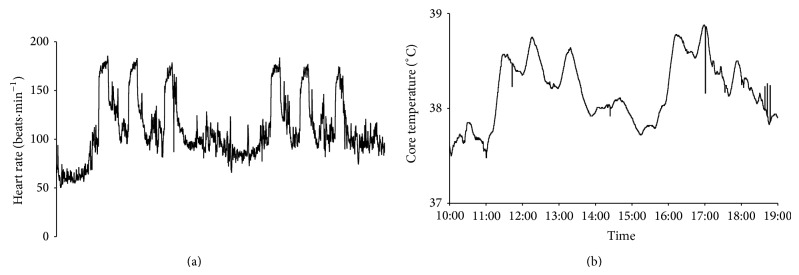
An example profile of heart rate (a) and core body temperature (b) for a representative driver during a single day of rally competition, with the six competitive special stages clearly visible in both profiles.

Average and peak HR and *T*_core_ were significantly higher during the rally than the reconnaissance (*P* = 0.012 to 0.018, *r* = 0.59 to 0.63), with drivers higher than codrivers during the rally but not the reconnaissance (Table 1). The 6 special stages elicited the highest responses in all drivers (Figure 1). Considerable intra- and interindividual variation occurred for *T*_sk_, with peak and average reconnaissance values significantly higher than rally (*P* = 0.012 and 0.017, *r* = 0.63 and 0.6, resp.) with little evidence of consistent differences between drivers and codrivers (Table 1).

### 3.3. Urine Osmolality and Fluid

**Figure 2:**
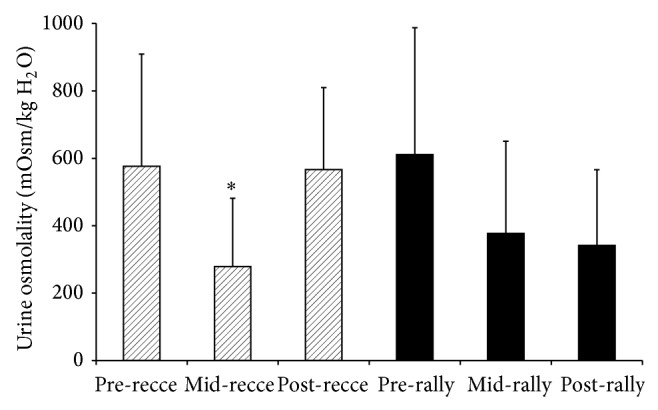
Mean (SD) values for urine osmolality of drivers and codrivers for the first morning sample (pre), during the day (mid) and at the end of one day of the reconnaissance (recce-hashed boxes) and then one day of the rally competition (solid black boxes). ^*^Significantly lower than postreconnaissance (*P* = 0.01).

*U*_osm_ data showed a significant main effect of time (*P* = 0.01, *r* = 0.62), although with large within participant (across time points) and between participant variations (Figure 2). The only significant increase was from mid- to postreconnaissance (*P* = 0.01), with other changes not significant (*P* = 0.44–1.0). Descriptive analysis shows 42% of driver samples at >700 mOsmol·kgH_2_O^−1^ (including two morning samples from the same participant >1000 mOsmol·kgH_2_O^−1^) compared to just 9% of codriver samples. Contrast case analysis across all measurements for the driver subgroup showed that the individual performer with highest *U*_osm_ drank the least volume on reconnaissance (2 L) and second least on rally (5.5 L) compared to the rest of the driver subgroup, and less than the whole group average for each day. In contrast the driver with lowest *U*_osm_ drank the second highest amount on reconnaissance (3.15 L) and most on rally (7.5 L) for the driver subgroup. Fluid intake for the whole group ranged from 1.75–4.75 L during the reconnaissance (8 h—equivalent to 0.39 L·h^−1^) and from 3–8.5 L during the rally (11 h—equivalent to 0.54 L·h^−1^).

### 3.4. Selective Attention

**Figure 3:**
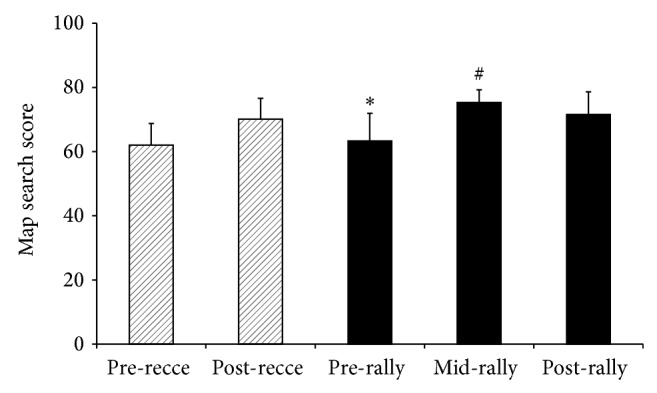
Mean (SD) values for scores of drivers and codrivers in the map search test of selective attention at the start of the day (pre), during the day (mid) and at the end of one day of the reconnaissance (recce-hashed boxes) and then one day of the rally competition (solid black boxes). ^*^Significantly lower than postreconnaissance (*P* = 0.007), mid- (*P* = 0.002) and postrally (*P* = 0.01); ^#^significantly higher than pre- (*P* = 0.002) and postreconnaissance (*P* = 0.01).

Attention scores on the map search task (Figure 3) showed a significant main effect of time (*P* < 0.001, *r* = 0.85), with lowest attention scores prereconnaissance and prerally although no significant difference between them (*P* > 0.99). Prerally attention was significantly lower than midrally (*P* = 0.002), postrally (*P* = 0.01) and postreconnaissance performance (*P* = 0.007). The highest attention level was midrally, significantly higher than both reconnaissance day performances (*P* = 0.002 and 0.01), but not significantly different to postrally (*P* = 0.401). There was no significant main effect of time on the auditory working memory task (*P* = 0.719, *r* = 0.26). This measure showed a tendency towards a ceiling effect.

## 4. Discussion

This field based investigation found important information about the physiological and cognitive responses of skilled performers operating under demanding conditions with high stakes. The key findings were that, as hypothesised, larger elevations in HR and *T*_core_ were recorded in response to competitive driving compared to noncompetitive, with values greater than would be predicted from simulated rally driving [2]. Drivers were also higher than codrivers. Consistent with the hypotheses there were significant changes in urine osmolality and attention. The driver subgroup produced more, high osmolality urine samples than codrivers, suggesting that they could be prone to dehydration. Specific case analysis found logical correspondence between urine osmolality and fluid intake. Attention scores were significantly lower at the start of the rally day than at other times during the day, but were not significantly different to the same time on the noncompetitive reconnaissance day.

Continuous measurement of HR and *T*_core_ provided accurate data on some of the physiological demands of rally at a major WRC event. Subgroup comparison revealed driver HR values were higher than codrivers during the competitive special stages as expected (Table 1, Figure 1), although across the whole day, mean values were similar. Importantly, driver HR values were significantly greater than during the reconnaissance, reflecting the additional psychophysiological demands of driving rally cars at high speeds, under competitive pressure and wearing protective clothing. For codrivers, there is additional incompetition cognitive load and arousal compared to reconnaissance, but only a small additional cardiovascular demand to maintain timing of pace-notes and body-position during high-speed turns, braking and jumps, evidenced by higher HR values in the special stages. The competitive stage driver HR values (Table 1), with peaks as high as 90% HR_max⁡_, are consistent with reported ranges in other driver studies [2–5]. The HR values, higher than previously published data for rally drivers during simulated driving in a heat chamber (mean 134 beats·min^−1^ at 50°C) [2], emphasize the need for research in real performance environments. Existing data on HR and energy expenditure suggest that although stages in rally are relatively short and intermittent, fatigue may occur during long, consecutive days which adds to the challenge of continually producing high level performance.

Rally *T*_core_ values in this study were significantly elevated compared to the noncompetitive reconnaissance conditions and slightly higher in drivers than codrivers (Table 1, Figure 1), with peak values during competitive stages similar to values reported in endurance competitions including yacht racing [14] and Ironman triathlon [13]. The competitors were successfully thermoregulating (e.g., *T*_core_ was recovering between stages with no accumulating increase) and avoiding hyperthermia, despite combined effects of radiant engine heat, physical exertion, and regulation safety clothing. Rally *T*_core_ values were slightly lower than previously reported for V8 drivers [4], probably due to the lower ambient (20.1°C versus 33.3°C) and in-car (33.9°C versus 48.6°C) temperatures. Ambient temperatures above 40°C are not unusual at some WRC events and it is logical to predict that *T*_core_ would be significantly higher for drivers in such events. With cumulative effects over multiple days drivers may be exposed to uncompensable heat storage and risk of hyperthermia with potential consequences for performance and safety [24, 25]. In addition the motivations involved in high level competition may override normal thresholds of comfort, as has been observed in motor sport [24]. Below extreme hyperthermia the potential consequences of high *T*_core_ on sports performance are currently unknown [9, 12–14, 25], but it would be expected that participants would at least experience increased perceived intensity and effort. Higher *T*_sk_ values during the reconnaissance (Table 1), without protective clothing or competitive driving forces, raises doubt on the accuracy of measuring *T*_sk_ from a single thermistor located in a chest-strap. Multiple site measurement is recommended [26], though was not possible during this rally.

Although it was not possible to definitively establish that performers were hypohydrated, given constraints of the study context and with a lack of an agreed gold standard for monitoring hydration status [19, 20, 27], some drivers exhibited *U*_osm_ values suggesting that they were competing in a potentially hypohydrated state (>700 mOsmol·kgH_2_O^−1^) [20]. In contrast, codrivers appeared to maintain euhydration better, presumably reflecting the reduced thermoregulatory challenge and possibly also better hydration strategies. One driver had *U*_osm_ first-morning values that were very high (>1000 mOsmol·kgH_2_O^−1^), these samples being most stable and reliable [20, 27] and interestingly this was also the driver who was consuming the least fluid during both days of monitoring. The driver consuming the most fluids maintained euhydration throughout, which underlines the importance of effective hydration strategies. Performers drank mostly water between stages and at service area where it was provided. The majority drank sports drinks containing electrolytes in-car, which could increase palatability, absorption, and prevent hyponatremia, under hot conditions that promote prolonged or excessive sweating, and limited food consumption. Fluid intakes reported here were similar to recommendations for marathon runners (0.4–1.0 L·h^−1^) [20], but there was high variability of drinking response and strategies, which is not surprising given interindividual variation in preferences [20, 28].

Prerally attention scores were significantly lower than mid- or postrally scores. Whilst this finding is consistent with the group differences in observed hydration states, care must be taken in interpreting this association given the reduced accuracy of urine osmolality in samples other than first-morning [20, 27]. However this pattern is consistent with literature on the impact of dehydration on cognitive function which has been found to occur at levels of dehydration equivalent to about 2% body mass loss [29, 30]. Low scores were found at the start of both days, compared to later day measures, and this can be explained by circadian rhythm led variation in cognitive performances [31] together with the enhancing effects of exercise on cognition [32]. It is noteworthy that despite the inevitable raised motivations for performers on the day of major competition, prerally attention performance was no better than prereconnaissance levels. Whilst the attention measures used in the current study had face validity with regard to task, and were robust to use in an extreme environment, the audio task may have been susceptible to a ceiling effect. Research on high performing individuals needs to address or avoid this limitation.

There are several practical applications that can be derived from the findings of this study that can be applied to rally performers and to individuals in comparable settings where they must combine motor skills and cognitive processing to complete tasks in demanding environments and with significant psychological pressure. The physiological demand, especially for drivers, shown in this study by elevated heart rate, emphasizes the need for good standards of physical fitness to prepare performers to meet demands effectively, reduce perceived effort, and enhance tolerance. Under relatively benign ambient temperature the effect of protective clothing and an increase in workload increased *T*_core_ significantly. In situations where ambient temperature is higher and there is greater restriction on fluid intake to aid cooling, the changes on *T*_core_ may be larger and reach levels where performance and safety might be compromised. Therefore specific actions to avoid unnecessary heat exposure and accumulation, and active engagement in cooling strategies, may become increasingly important. Low attention at the start of the rally day despite motivation may result in detrimental cognitive processing. Morning team briefings are common in rally sport and other situations that require shared understanding (police, military, and medicine) when attention of performers is compromised by time of day (or other factors such as fatigue). Therefore, attempts should be made to offset this decrement (e.g., exercise) or to facilitate processing (mnemonics, cues, and reduced information load).

This study is the first to our knowledge to publish field based group data on rally driver/codriver responses, providing a contrast between reconnaissance and rally days. The wireless monitoring systems and access to performers through the duration of the rally provide a rare opportunity to examine the responses in real life context. The physiological responses and cognitive performance of performers under extreme conditions is of interest for both applied and research perspectives. Practical application can assist performers to maintain standards and safety despite additional challenges of the environment. For researchers, understanding what happens in real performance contexts is critical to ensure that concepts and ideas formulated in controlled research situations with volunteer, mostly student participants, are truly representative and have adequate ecological validity.

## 5. Conclusions

This study has demonstrated the extent of heart rate elevation within competitive rally driving providing a clear indication of both the peak level (in special stages) and the prolonged lower level elevation across a performance day. Temperature also rose but the elevation was easily tolerable in the ambient temperature during this study. The performers reported a very wide range of fluid intake and urine osmolality despite operating in the same team and receiving the same education on this issue. This reflects the difficulty of ensuring that scientific knowledge is applied effectively to ensure optimal performance state. Cognitive performance followed a pattern consistent with circadian rhythm and was not increased at the start of the event, even though this was of very high importance. Understanding these responses and providing information and strategies to minimise any detrimental impact would be beneficial to those involved in a range of high performance situations.

## Figures and Tables

**Table 1:** Summary data (peak and mean) during reconnaissance and rally, for heart rate (HR, including values during the competitive special stages (SS) only), core temperature (*T*_core_), and skin temperature (*T*_sk_). Data are shown as means (SD) for the whole group (in bold) and also separately for drivers and codrivers.

	Rally			Reconnaissance		
	Group (*n* = 8)	Drivers (*n* = 4)	Codrivers (*n* = 4)	Group (*n* = 10)	Drivers (*n* = 5)	Codrivers (*n* = 5)
SS HR (beats·min^−1^)	**133 (24)**	154 (12)	113 (12)	—	—	—
Mean HR (beats·min^−1^)	109^*^ **(8)**	113 (11)	105 (11)	**82 (12)**	80 (16)	84 (16)
Peak HR (beats·min^−1^)	166^*^ **(17)**	177 (18)	155 (18)	**111 (16)**	106 (19)	116 (19)
Mean *T*_core_ (°C)	37.9^*^ **(0.3)**	38.1 (0.4)	37.8 (0.4)	**37.3 (0.2)**	37.3 (0.3)	37.4 (0.3)
Peak *T*_core_ (°C)	38.5^*^ **(0.4)**	38.8 (0.4)	38.2 (0.4)	**37.6 (0.2)**	37.7 (0.3)	37.6 (0.3)
Mean *T*_sk_ (°C)	33.9^#^ **(0.5)**	33.7 (0.4)	34.1 (0.4)	**35.0 (0.6)**	35.0 (0.7)	35.0 (0.7)
Peak *T*_sk_ (°C)	36.7^#^ **(0.6)**	37.0 (0.2)	36.4 (0.2)	**38.1 (1.4)**	38.0 (1.7)	38.3 (1.7)

^*^Significantly greater than reconnaissance values (*P* = 0.012 to 0.018).

^#^Significantly lower than reconnaissance values (*P* = 0.012 to 0.017).

## References

[1] Watkins E. S. (2006). The physiology and pathology of formula one Grand Prix motor racing. *Clinical Neurosurgery*.

[2] Walker S. M., Ackland T. R., Dawson B. (2001). The combined effect of heat and carbon monoxide on the performance of motorsport athletes. *Comparative Biochemistry and Physiology Part A: Molecular & Integrative Physiology*.

[3] Jacobs P. L., Olvey S. E., Johnson B. M., Cohn K. A. (2002). Physiological responses to high-speed, open-wheel racecar driving. *Medicine and Science in Sports and Exercise*.

[4] Brearley M. B., Finn J. P. (2007). Responses of motor-sport athletes to v8 supercar racing in hot conditions. *International Journal of Sports Physiology and Performance*.

[5] Schwaberger G. (1987). Heart rate, metabolic and hormonal responses to maximal psycho-emotional and physical stress in motor car racing drivers. *International Archives of Occupational and Environmental Health*.

[6] Beaune B., Durand S., Mariot J.-P. (2010). Open-wheel race car driving: energy cost for pilots. *Journal of Strength and Conditioning Research*.

[7] Lieberman H. R. (2007). Hydration and cognition: a critical review and recommendations for future research. *Journal of the American College of Nutrition*.

[8] Morley J., Beauchamp G., Suyama J. (2012). Cognitive function following treadmill exercise in thermal protective clothing. *European Journal of Applied Physiology*.

[9] Dugas J. P. (2010). How hot is too hot?: some considerations regarding temperature and performance. *International Journal of Sports Physiology and Performance*.

[10] Goulet E. D. B. (2013). Effect of exercise-induced dehydration on endurance performance: evaluating the impact of exercise protocols on outcomes using a meta-analytic procedure. *British Journal of Sports Medicine*.

[11] Sawka M. N., Noakes T. D. (2007). Does dehydration impair exercise performance?. *Medicine and Science in Sports and Exercise*.

[12] Schlader Z. J., Stannard S. R., Mündel T. (2011). Exercise and heat stress: Performance, fatigue and exhaustion—a hot topic. *British Journal of Sports Medicine*.

[13] Laursen P. B., Suriano R., Quod M. J. (2006). Core temperature and hydration status during an Ironman triathlon. *British Journal of Sports Medicine*.

[14] Neville V., Gant N., Folland J. P. (2010). Thermoregulatory demands of elite professional America's Cup yacht racing. *Scandinavian Journal of Medicine & Science in Sports*.

[15] Armstrong L. E., Ganio M. S., Casa D. J. (2012). Mild dehydration affects mood in healthy young women. *Journal of Nutrition*.

[16] Ganio M. S., Armstrong L. E., Casa D. J. (2011). Mild dehydration impairs cognitive performance and mood of men. *British Journal of Nutrition*.

[17] Roberts A. P. J., Cole J. C. (2013). The effects of exercise and body armor on cognitive function in healthy volunteers. *Military Medicine*.

[18] Byrne C., Lim C. L. (2007). The ingestible telemetric body core temperature sensor: a review of validity and exercise applications. *British Journal of Sports Medicine*.

[19] Armstrong L. E. (2007). Assessing hydration status: the elusive gold standard. *Journal of the American College of Nutrition*.

[20] Sawka M. N., Burke L. M., Eichner E. R., Maughan R. J., Montain S. J., Stachenfeld N. S. (2007). Exercise and fluid replacement. *Medicine and Science in Sports and Exercise*.

[21] Robertson I. H., Ward T., Ridgeway V., Nimmo-Smith I. (1994). *The Test of Everyday Attention—Manual*.

[22] Leach J., Ansell L. (2008). Impairment in attentional processing in a field survival environment. *Applied Cognitive Psychology*.

[23] Hopkins W. G. A Scale of Magnitudes for Effect Statistics. http://sportsci.org/resource/stats/.

[24] Jareño A., de la Serna J. L., Cercas A., Lobato A., Uyá A. (1987). Heat stroke in motor car racing drivers. *British Journal of Sports Medicine*.

[25] Armstrong L. E., Casa D. J., Millard-Stafford M., Moran D. S., Pyne S. W., Roberts W. O. (2007). Exertional heat illness during training and competition. *Medicine and Science in Sports and Exercise*.

[26] Sawka M. N., Latzka W. A., Montain S. J. (2001). Physiologic tolerance to uncompensable heat: intermittent exercise, field vs laboratory. *Medicine and Science in Sports and Exercise*.

[27] Shirreffs S. M., Maughan R. J. (1998). Urine osmolality and conductivity as indices of hydration status in athletes in the heat. *Medicine and Science in Sports and Exercise*.

[28] Maughan R. J., Shirreffs S. M. (2008). Development of individual hydration strategies for athletes. *International Journal of Sport Nutrition and Exercise Metabolism*.

[29] Cian C., Barraud P. A., Melin B., Raphel C. (2001). Effects of fluid ingestion on cognitive function after heat stress or exercise-induced dehydration. *International Journal of Psychophysiology*.

[30] Grandjean A. C., Grandjean N. R. (2007). Dehydration and cognitive performance. *Journal of the American College of Nutrition*.

[31] Schmidt C., Collette F., Cajochen C., Peigneux P. (2007). A time to think: circadian rhythms in human cognition. *Cognitive Neuropsychology*.

[32] McMorris T., Sproule J., Turner A., Hale B. J. (2011). Acute, intermediate intensity exercise, and speed and accuracy in working memory tasks: a meta-analytical comparison of effects. *Physiology & Behavior*.

